# GX-I7, a long-acting IL-7, safely and effectively increased peripheral CD8^+^/CD4^+^ T cells and TILs in patients with locally advanced or metastatic solid tumours

**DOI:** 10.1038/s41416-025-03069-3

**Published:** 2025-06-09

**Authors:** Gun Min Kim, Sojeong Kim, Myung Ah Lee, Mi-Sun Byun, Donghoon Choi, Se Hwan Yang, JungWon Woo, Young Chul Sung, Eui-Cheol Shin, Su-Hyung Park, Tae Won Kim, Joohyuk Sohn

**Affiliations:** 1 Division of Medical Oncology, Department of Internal Medicine, Yonsei Cancer Center, Yonsei University College of Medicine, Seoul, Korea; 2 Graduate School of Medical Science and Engineering, Korea Advanced Institute of Science and Technology, Daejeon, Korea; 3 Division of Hematology, Department of Internal Medicine, Yonsei University College of Medicine, Seoul, Republic of Korea; 4 Division of Medical Oncology, Seoul St, Mary’s Hospital, The catholic University of Korea, Seoul, Korea; 5 Genexine, Inc., Seoul, Korea; 6 NeoImmuneTech, Inc., Rockville, MD USA; 7 Department of Oncology, University of Ulsan College of Medicine, Asan Medical Center, Seoul, Korea

**Keywords:** Cancer, Drug development, Phase II trials

## Abstract

**Background:**

GX-I7 (rhIL-7-hyFc, efineptakin alfa) is a hybrid Fc-fused long-acting interleukin-7 (IL-7) with the aim of correcting T-cell deficiency, thereby strengthening the immune response to fight against cancer. This Phase 1b, dose-escalation study was designed to assess the safety, tolerability, pharmacokinetics (PK), and pharmacodynamics (PD) of GX-I7 in patients with locally advanced or metastatic solid tumours.

**Methods:**

This study, conducted in patients with advanced solid tumours at three hospitals in Korea, involved intramuscular GX-I7 administration across eight dose levels (60–1700 µg/kg) in 3- and 6-week cohorts. A dose-expansion phase at 720 and 1200 µg/kg further assessed GX-I7’s safety and efficacy.

**Results:**

Anti-tumour responses showed either stable disease (SD) or disease progression (PD). GX-I7 demonstrated dose-dependent increases in the maximum serum concentration (C_max_) and area under the curve up to the last measurable concentration (AUC_last_). In addition, a dose-dependent increase in circulating CD8^+^/CD4^+^ T cells was observed. In five patients who consented for biopsy, a statistically significant increase in tumour-infiltrating lymphocytes (TILs) followed GX-I7 treatment.

**Discussion:**

Findings suggest GX-I7 is a safe T cell-amplifying agent with peripheral immune activation. Ongoing studies are exploring its ability to enhance immune responses in peripheral immune cells and tumour cells when combined with other anti-cancer agents.

**Clinical Trial registration:**

NCT03478995

## Introduction

Surgery and chemotherapy have long been the two major modalities for cancer treatment, while chemotherapy is the sole option for patients with non-resectable advanced or metastatic solid tumours. However, with the emergence of immune therapies, such as immune checkpoint inhibitors (ICI), T cells have become a central focus in engaging the immune system in the battle against cancer [1]. Previous studies have revealed that lymphopenia [2], including anti-cancer treatment-induced lymphopenia, is associated with inferior survival outcomes [3–5]. Furthermore, a low response to ICI therapy has been observed in patients with low lymphocyte count [6]. As T cells play a central role in the immune response to cancer, correcting lymphopenia and increasing tumour-infiltrating lymphocytes (TILs) are crucial in enhancing the immune response to cancer, thereby improving treatment outcomes [7, 8].

GX-I7 (rhIL-7-hyFc, efineptakin alfa) is a hybrid Fc-fused long-acting recombinant human interleukin-7 (IL-7) developed by Genexine, Inc., with the aim of correcting T cell deficiency, thereby strengthening the immune response to fight cancer and creating a favourable environment for anti-cancer treatment or ICIs. IL-7 is a homoeostatic cytokine that plays an important role in the development, maintenance, proliferation, and regeneration of T-cells [9, 10]. Despite the clinical benefits of IL-7 in various therapeutic areas, including cancer, the development of IL-7 as a commercial drug is limited by its short half-life [11–19].

GX-I7 effectively lengthens the IL-7 half-life by six times compared to that of rhIL-7 via neonatal Fc receptor (FcRn)-mediated recycling [20, 21]. In vivo, GX-I7 led to an increase in peripheral lymphocyte counts and TILs when administered either in monotherapy or combination therapy with cyclophosphamide (CPA) or ICI (e.g., anti-PD-(L)1 or anti-CTLA-4 antibody), and showed anti-tumour efficacy in murine syngeneic tumour models [22].

With in vivo and clinical Phase 1 study results in healthy volunteers supporting further development of GX-I7 to correct T cell deficiencies, this study was designed to assess safety, tolerability, pharmacokinetics (PK) and pharmacodynamics (PD) of GX-I7 in patients with locally advanced or metastatic solid tumours.

## Materials and Methods

### Ethics statement

This study was approved by the institutional review board of each research site and conducted in full accordance with the Declaration of Helsinki and Korean Good Clinical Practice (www.clinicaltrials.gov registration number: NCT03478995). Written informed consent was obtained from all patients prior to conducting any study-related procedures.

### Study design

This study was a Phase 1b, open-label, dose-escalation study to evaluate the safety, tolerability, PK, and PD of GX-I7 in patients with locally advanced or metastatic solid tumours. The research was conducted across three medical centres: Severance Hospital, ASAN Medical Centre, and Seoul ST. Mary’s Hospital. Enrolment commenced on March 5, 2018 with the inclusion of the first subject, and the trial concluded on March 16, 2020, upon completion of the last subject. The primary objectives were to determine the recommended Phase 2 dose (RP2D) and maximum tolerated dose (MTD) and to characterize dose-limiting toxicities (DLTs). The classic 3 + 3 design was adopted for sequential dose escalation, and the DLT evaluation period was 3 weeks. The number of subjects was up to 35, with 3–12 subjects per dose group. The total number of enrolled subjects was adjusted based on the results of the dose escalation phase and the number of dose groups to be evaluated during the dose expansion phase.

### Patients

Eligible patients were aged ≥19 years and had an Eastern Cooperative Oncology Group (ECOG) performance status of 0 or 1, an estimated life expectancy of >12 weeks, and appropriate haematologic, hepatic, and end-organ function values. Patients had histological documentation of locally advanced, recurrent, or metastatic incurable solid tumours that had failed to respond to known or available therapy, or for whom standard therapy was considered inappropriate. Exclusion criteria included any anti-tumour therapy within three weeks and previous use of an ICI or immunomodulatory monoclonal antibody (mAb)-derived drug within 12 weeks prior to the initiation of study treatment.

### Treatment

Patients were enroled to receive GX-I7 intramuscularly at three- or six-week intervals. The dose was escalated, and a total of eight dose levels were tested, each with at least three patients, at doses of 60, 120, 240, 480, 720, 960, 1200, and 1700 µg/kg at three-week intervals. Out of the eight tested dose levels, two (720 and 1200 µg/kg) were selected as potential RP2D for expansion, and up to six additional patients were enroled at these doses for the expansion phase. At a dose expansion of 1200 µg/kg, the efficacy of GX-I7 was evaluated at both three- and six-week intervals. For biopsy-consented patients, tumour tissue was collected both pre and three–five weeks after post-GX-I7 administration to test changes in TILs.

### Safety, tolerability, and immunogenicity assessments

The safety and tolerability of GX-I7 were assessed throughout the study by adverse event (AE) monitoring using the NCI Common Terminology Criteria for Adverse Events (CTCAE) (version 4.03). Undesirable symptoms, clinically significant abnormalities in clinical laboratory tests, vital signs, physical examination, or DLT, as well as the emergence of antibodies (anti-drug antibody [ADA] and neutralizing antibody [nAb]) were included. Treatment-emergent adverse events (TEAEs) were analysed. The presence of antibodies against GX-I7 was determined using a validated enzyme-linked immunosorbent assay (ELISA), as described in the GX-I7 Phase 1 First-in-human (FIH) study [23]. All patients who tested positive for ADA were analysed for its neutralizing property (nAb).

### Anti-tumour efficacy

Solid tumour assessments were performed by CT scan or MRI every 2 cycles up to cycle 8, and every four cycles or as clinically indicated thereafter. Tumour response and progression were evaluated according to the Response Evaluation Criteria in Solid Tumours (RECIST) (version 1.1).

### PK analysis

Blood samples for PK analysis were collected during Cycle 1 while the patients were hospitalized at various time points, including pre-dose and 0.5, 6, 12, 24, 48, and 72 hours post-dose. Additional blood collections were performed on Cycle 1 Day 8 (C1D8), C1D15, and C2D1 for patients administered GX-I7 every 3 weeks and C1D8, C1D15, C1D22, and C2D1 for patients administered GX-I7 every 6 weeks. Serum IL-7 concentrations were measured using the Quantikine HS ELISA Human IL-7 immunoassay kit (R&D Systems, Minneapolis, MN), and the detailed analysis method was based on a previously reported Phase 1 study [22]. The PK parameters were derived by a non-compartmental method using Phoenix WinNonlin software (version 7.0, Certara, St. Louis, MO, USA). In addition, the correlation between the administered dose and AUC_last_ and C_max_ was analysed.

### PD analysis

The PD variables were changes in absolute lymphocyte counts (ALC) and subsets of immune cells (T, B, and NK cells) in the blood assessed on a weekly basis at pre-dose, C1D1, and C2D1. Thereafter, it was performed on day 1 (pre-dose) of every cycle and at the treatment discontinuation (DC) visit. Absolute T, B, and NK cell counts were calculated using ALCs multiplied by the proportions of CD3^+^, CD19^+^ and CD56^+^ cells against total lymphocytes, and absolute CD4^+^ and CD8^+^ T cells were calculated using ALCs multiplied by the proportions of CD4^+^ and CD8^+^ T cells against total CD3^+^ T cells, respectively. Ki67^+^ and CD127^+^ T cell counts were calculated by multiplying the proportion of Ki67^+^-or CD127^+^-expressing cells by total CD4^+^ and CD8^+^ T cells, respectively. All analyses were performed using multicolour flow cytometry. The following anti-human fluorochrome-conjugated antibodies were used: anti-CD3- Alexa Fluor 700 (UCHT1), anti-IL-7Rα-BV421 (A019D5), anti-CD4-PE-Cy5 (OKT4), anti-CD45RA-APC-Cy7 (HI100), anti-CCR7-BV785 (G043H7), anti-CD25-BV650 (BD96; BioLegend), anti-CD19-BV711 (SJ25C1), anti-CD14-BV711 (MφP9), anti-CD8-Alexa Fluor 700 (RPA-T8; all from BD Biosciences), and anti-CD56-APC (CMSSB; Thermo Fisher). Simultaneously, dead cells were stained using a Live/Dead Fixable Red Dead Cell Stain Kit (Thermo Fisher Scientific). The cells were fixed and permeabilized using a Foxp3 Staining Buffer Set (Thermo Fisher), and intracellular molecules were stained (4 °C, 20 min) using anti-Ki-67-PE-Cy7 (20Raj1) and anti-Foxp3-PE (236A/E7; Thermo Fisher) (Supplementary material). To calculate the maximum ALC and the area under the ALC-time curve until the last sampling time, the Phoenix WinNonlin software was used after correcting the baseline values. The change in TILs at pre and post GX-I7 administration was evaluated in patients who consented to biopsy using Multiplex IHC analysis (Supplementary Material).

### Statistical analysis

All demographic characteristics and PK/PD parameters were summarized using descriptive statistics. Continuous variables were summarized using the number of observations, mean, standard deviation, coefficient of variation, median, and range as appropriate. Categorical values were summarized using the number of observations and percentages as appropriate. Data were summarized for the dose cohort, expansion cohort, and total patient population. All assessments before the first dose of the study drug were considered as baseline. If multiple baseline assessments were performed, the most recent assessment was used for statistical analysis. A parametric or nonparametric method was used based on the results of the normality test. All analyses were 2-sided, with a significance level of 5%, and a 95% confidence interval (CI) was calculated. All statistical analyses were performed using SAS® Version 9.4 (SAS Institute, Cary, NC, USA).

## Results

### Patients disposition and baseline characteristics

**Fig. 1:**
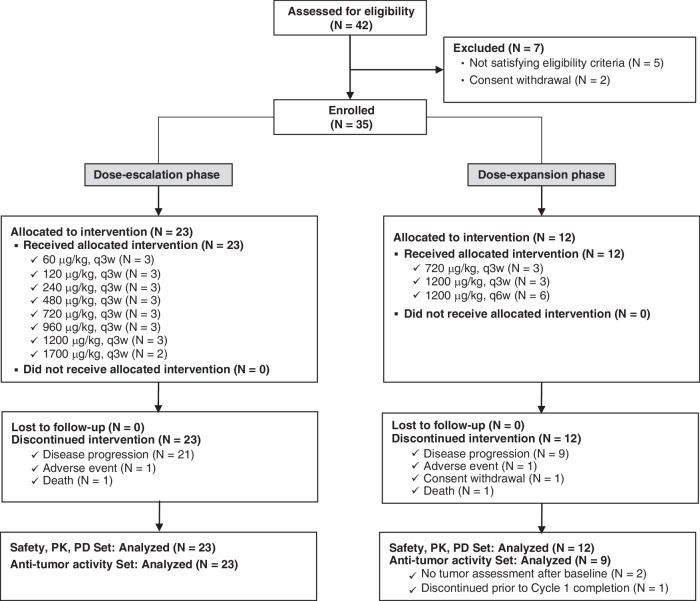
Patient disposition. Patients in the dose-escalation stage were treated with 60, 120, 240, 480, 720, 960, 1200 and 1700 µg/kg of GX-I7 intramuscularly every 3 weeks (Q3W). In the dose-expansion stage, patients received a 720 and 1200 µg/kg dose of GX-I7 intramuscularly every 3 weeks (Q3W) or every 6 weeks (Q6W), which were selected in the dose-escalation stage.

Out of 42 patients screened across three hospitals, 35 patients were enroled in the study and received at least one dose of GX-I7, with seven patients being excluded because they either did not satisfy the eligibility criteria (five patients) or withdrew their consent (two patients). In the dose escalation Phase, according to the classical 3 + 3 design, 3 patients were assigned to each group. In the 1700 µg/kg group, only two patients were enroled because of the occurrence of one DLT. The group receiving 720 µg/kg, which showed the highest increase in ALC fold change, and 1200 µg/kg as the maximum tolerated dose (MTD) was selected as the potential recommended Phase 2 dose (RP2D). Three patients receiving 720 µg/kg group every 3 weeks (q3w), three patients receiving 1200 µg/kg q3w, and six patients receiving 1200 µg/kg q6w were enroled in the dose expansion (Fig. 1). A dose expansion group receiving GX-I7 q6w was included after determining that since the ALC remained elevated at the time of the q3w repeated dosing, it was not expected that frequent administration of GX-I7 would bring additional benefits. The median age (range) was 58 (40–75) years; 54% were male, and all were Asian. Baseline ECOG performance status was 0 in 49% of patients, and the most common site of the primary tumour was colon/rectal in 66% of patients, followed by the breast in 11%, and the ovary in 9% of patients. At the time of study enrolment, metastatic lesions were commonly found in the lungs (32 patients), followed by the liver (24 patients), and lymph nodes (14 patients). All subjects enroled in the study were at stage IV with a third or more lines of treatment. In previous therapies, the majority of subjects had been treated with antimetabolite, targeted, and platinum-based agents. The subjects discontinued their previous treatment primarily because of disease progression (PD). The demographic and baseline characteristics are summarized in Table 1.

**Table 1:** Baseline characteristics.

Group	GX-I7 60 µg/kg (*n* = 3)	GX-I7 120 µg/kg (*n* = 3)	GX-I7240 µg/kg (*n* = 3)	GX-I7 480 µg/kg (*n* = 3)	GX-I7 720 µg/kg (*n* = 6)	GX-I7 960 µg/kg (*n* = 3)	GX-I7 1200 µg/kg (*n* = 12)	GX-I7 1700 µg/kg (*n* = 2)	Total (*n* = 35)
Age (years)									
Median (Min, Max)	58 (52, 64)	58 (55, 58)	59 (52, 75)	44 (44, 48)	57 (45, 71)	58 (45, 65)	62 (43, 75)	54 (48, 60)	58 (40, 75)
Gender, *N* (%)									
Male	2 (67)	0	3 (100)	2 (67)	4 (67)	1 (33)	6 (50)	1 (50)	19 (54)
Race, *N* (%)									
Asian	3 (100)	3 (100)	3 (100)	3 (100)	6 (100)	3 (100)	12 (100)	2 (100)	35 (100)
ECOG performance status, *N* >(%)									
0	1 (33)	1 (33)	1 (33)	0	5 (83)	2 (67)	5 (42)	2 (100)	17 (49)
1	2 (67)	2 (67)	2 (67)	3 (100)	1 (17)	1 (33)	7 (58)	0	18 (51)
Site of Primary Tumour, *N* (%)									
Breast	1 (33)	0	0	1 (33)	0	0	2 (17)	0	4 (11)
Colon/Rectal	1 (33)	2 (67)	2 (67)	2 (67)	4 (67)	3 (100)	7 (58)	2 (100)	23 (66)
Ovary	0	1 (33)	0	0	0	0	2 (17)	0	3 (9)
Other	1 (33)	0	1 (33)	0	2 (33)	0	1 (8)	0	5 (14)
Location of Metastatic, *N*									
Abdominal Wall	0	1	0	0	1	1	1	0	4
Adrenal Gland	1	0	1	0	0	0	1	0	3
Bone	1	0	0	1	1	0	1	0	4
Brain	0	0	0	1	0	0	1	0	2
Liver	2	2	2	2	1	3	10	2	24
Lung	3	2	3	3	5	3	11	2	32
Lymph Node	2	2	1	2	3	2	2	0	14
Pleura	1	0	0	0	0	0	1	0	2
Other	2	0	2	1	2	2	4	0	13
Previous illness of systemic therapy, *N* (%)									
0	0	0	0	0	0	0	1 (8)^†^	0	1 (3)
1	0	0	0	0	0	0	0	0	0
2	0	0	1 (33)	1 (33)	1 (17)	0	1 (8)	0	4 (11)
3	1 (33)	0	0	0	4 (67)	1(33)	5 (42)	2 (100)	13 (37)
≥4	2 (67)	3 (100)	2 (67)	2 (67)	1 (17)	2 (67)	5 (42)	0	17 (49)

*ECOG PS* Eastern Cooperative Oncology Group performance status.

†Patient received chemotherapy for adjuvant.

### Safety

The safety analysis set included patients who received at least one dose of GX-I7 during the clinical trial. Safety data from all 35 enroled patients were used to assess the safety profiles. GX-I7 intramuscular administration was safe and well tolerated across doses ranging from 60 to 1200 µg/kg, except for the 1700 µg/kg dose. A total of 225 TEAEs occurred in all the 35 patients (100%). Among these, 116 adverse drug reactions (ADRs) occurred in 28 of 35 patients (80.0%), with the majority rated as Grade 1 and 2 (68 and 43 cases, respectively). The most frequently reported ADR was injection site reaction (61 cases in 25 patients (71.4%)), followed by pyrexia with 21 cases in 12 patients (34.3%), then rash with 8 cases in 7 patients (20.0%). All 61 reported injection site reactions were classified as Grade 1 or 2, with most requiring medications for resolution, while many resolved spontaneously.

In the 720, 1200, and 1700 μg/kg groups, grade 3 or higher ADRs were reported: fatigue (720 and 1200 µg/kg), hypersensitivity, and anaphylactic reactions (1700 μg/kg). Due to grade 3 fatigue and anaphylactic reaction, two patients discontinued GX-I7 permanently. No GX-I7 related death have been reported.

The dose-limiting toxicity of GX-I7 was assessed in 35 patients for all dose groups, and at 1700 μg/kg, one out of two patients experienced DLT, which was a case of Grade 3 or higher hypersensitivity. Therefore, the MTD was determined to be 1200 μg/kg, and this dose administered every 6 weeks was selected as RP2D. Regardless of the administration interval, the incidence of ADA was observed, however, no ADA-related AE were observed. A summary of the safety profile is presented in Table 2.

**Table 2:** Summary of safety profiles.

Group	GX-I760 µg/kg (*n* = 3)	GX-I7120 µg/kg (*n* = 3)	GX-I7240 µg/kg (*n* = 3)	GX-I7 480 µg/kg (*n* = 3)	GX-I7 720 µg/kg (*n* = 6)	GX-I7 960 µg/kg (*n* = 3)	GX-I7 1200 µg/kg (*n* = 12)	GX-I7 1700 µg/kg (*n* = 2)	Total (*n* = 35)
Subjects with Treatment-Emergent Adverse Events, *n* (%) [Events]									
	3 (100.0) [14]	3 (100.0) [30]	3 (100.0) [15]	3 (100.0) [21]	6 (100.0) [35]	3 (100.0) [6]	12 (100.0) [88]	2 (100.0) [16]	35 (100.0) [225]
Subject with Adverse Drug Reactions, *n* (%) [Events]									
	3 (100.0) [6]	2 (66.7) [9]	3 (100) [6]	2 (66.7) [8]	4 (66.7) [18]	2 (66.7) [4]	10 (83.3) [49]	2 (100.0) [16]	28 (80.0) [116]
General disorders and administration site conditions									
Injection site reaction	3 (100.0) [6]	2 (66.7) [5]	3 (100.0) [3]	2 (66.7) [6]	3 (50.0) [9]	1 (33.3) [3]	9 (75.0) [24]	2 (100.0) [5]	25 (71.4) [61]
Grade 1	2(66.7)		1(33.3)	1(33.3)	1(16.7)		4(33.3)	1(50.0)	
Grade 2	1(33.3)	2(66.7)	2(66.7)	1(33.3)	2(33.3)	1(33.3)	5(41.7)	1(50.0)	
Pyrexia	0	1 (33.3) [1]	1 (33.3) [1]	1 (33.3) [2]	1 (16.7) [2]	1 (33.3) [1]	5 (41.7) [11]	2 (100.0) [3]	12 (34.3) [21]
Grade 1		1(33.3)	1(33.3)	1(33.3)		1(33.3)	5(41.7)	1(50.0)	
Grade 2								1(50.0)	
Fatigue	0	0	0	0	1 (16.7) [1]	0	3 (25.0) [3]	0	4 (11.4) [4]
Grade 1							1(8.3)		
Grade 2							1(8.3)		
Grade 3					1(16.7)		1(8.3)		
Skin and subcutaneous tissue disorders									
Rash	0	0	0	0	3 (50.0) [4]	0	2 (16.7) [2]	2 (100.0) [2]	7 (20.0) [8]
Grade 1							1(8.3)	1(50.0)	
Grade 2					3(50.0)		1(8.3)	1(50.0)	
Rash popular	0	1 (33.3) [1]	0	0	0	0	0	0	1 (2.9) [1]
Grade 1		1(33.3)							
Immune system disorders									
Anaphylactic reaction	0	0	0	0	0	0	0	1 (50.0) [1]	1 (2.9) [1]
Grade 3								1(50.0)	
Hypersensitivity	0	0	0	0	0	0	0	1 (50.0) [1]	1 (2.9) [1]
Grade 3								1(50.0)	
Gastrointestinal disorders									
Nausea	0	0	0	0	0	0	1 (8.3) [1]	0	1 (2.9) [1]
Grade 2							1(8.3)		
Investigations									
Platelet count decreased	0	0	0	0	0	0	0	1 (50.0) [1]	1 (2.9) [1]
Grade 2								1(50.0)	

### Tumour response

Anti-tumour activity was analysed for 32 patients who completed one cycle of the treatment and measurable disease among those who received at least one dose of GX-I7. There were no complete response (CR) or partial response (PR) observed when assessed by RECIST version 1.1. The objective response rate (ORR) was 0%, as all responses showed either stable disease (SD) or disease progression (PD). Six patients (18.75%) showed disease control as stable disease (SD) and 26 patients (81.25%) showed disease progression (PD). Among these six patients, the doses received were 60, 120, 240, 720, and 1200 µg/kg, as shown in Supplementary Table S1. The median PFS was evaluated for 31 patients, excluding one patient whose tumour evaluation result was stable disease (SD) among 32 patients with anti-tumour activity set. The median PFS was 5.69 weeks (95% CI; 5.13, 5.98), showing similar PFS across dose groups.

### Pharmacokinetics (PK)

**Fig. 2:**
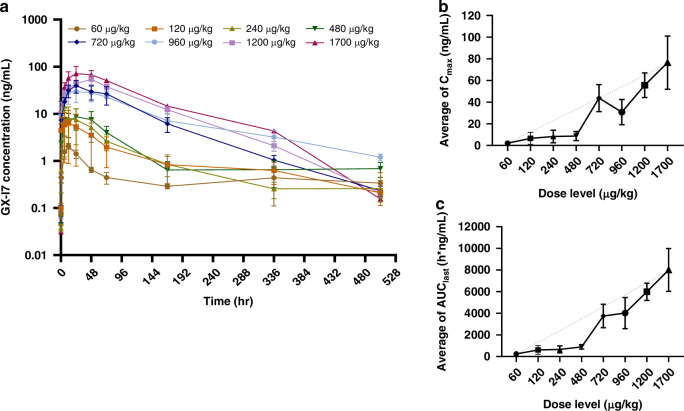
Pharmacokinetics profiles following the first intramuscular administration of GX-I7 ranging from 60 to 1700 μg/kg. **a** The mean serum concentration-time profiles of GX-I7, **b** the maximum concentration (C_max_) versus dose of GX-I7, and **C** the area under the serum concentration-time curve from time zero to the last measurable time-point (AUC_last_) versus dose of GX-I7. The results are presented as mean ± standard deviation (SD).

This study conducted a PK analysis of 35 patients from all cohorts. As shown in Fig. 2a, serum concentration was measured at various time points after intramuscular administration of GX-I7, ranging from 60 to 1700 μg/kg. The PK profile of GX-I7 showed a dose-dependent response. The peak serum concentration (C_max_) was reached at 11-47.5 hours post dosing, with peak serum concentration ranging from 2.11 to 76.5 ng/mL, and a half-life lasting from 60.8 to 139.7 hours (Supplementary Table S2). AUC_last_ and C_max_ increased linearly with dose (R^2^ = 0.9174 and 0.9657, respectively; Fig. 2b, c).

### Pharmacodynamics (PD)

**Fig. 3:**
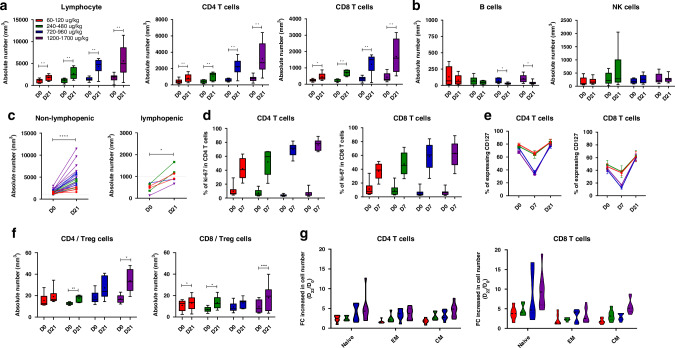
Pharmacodynamic profiles of GX-I7 following the first intramuscular administration across different doses groups. **a** Changes in absolute lymphocyte count (ALC), CD4^+^, and CD8^+^ T cells, **b** Changes in other immune cells, **c** ALC in non-lymphopenic (ALC 1000 cells/mm^3^) and lymphopenic (ALC 1000 cells/mm^3^) patients, **d** Percentage (%) of Ki67^+^CD4^+^ and Ki67^+^CD8^+^ T cells,^,^ **e** Percentage (%) of CD127^+^CD4^+^ and CD127^+^CD8^+^ T cells, **f** CD4^+^T/Treg and CD8^+^T/Treg cell ratios and **g** Subsets of CD4^+^ and CD8^+^ T cells. *p < 0.05, **p < 0.01, ***p < 0.001 versus baseline (Day 0) by Wilcoxon matched-pairs signed rank test. FC fold change.

PD analyses were performed for changes in the absolute lymphocyte count (ALC) and lymphocyte subsets. The ALC, CD4^+^ and CD8^+^ T cell counts showed a statistically significant increase at C1D21 compared to the baseline at all dose levels:60-120, 240-480, 720-960 and 1200-1700 µg/kg groups (*p < 0.05, **p < 0.01, ***p < 0.001, Fig. 3a) in a dose-dependent manner. In contrast, GX-I7 administration did not cause significant changes in B or NK cells (Fig. 3b). These results are consistent with previously reported findings [23]. Increases in ALC were observed in both lymphopenic and non-lymphopenic patients (*p < 0.05, ****p < 0.0001, respectively; Fig. 3c).

To evaluate the potential mechanism by which GX-I7 increases lymphocyte counts, Ki67^+^CD4^+^ T cells and Ki67^+^ CD8^+^ T cells were measured. Their percentage peaked on day 7 after treatment and showed a dose-dependent and statistically significant increase in all the groups (Fig. 3d). IL-7 binds to IL-7 receptor α (CD127) expressed on the surface of T cells, and CD127 is downregulated or internalized when T cells are over-activated by IL-7 (transcytosis). Indeed, there was a significant but transient decrease in CD127^+^CD4^+^ and CD127^+^ CD8^+^ T cells on day 7 compared with day 0 in each group. However, these decreases were restored at three weeks post-dose (Fig. 3e). Previous clinical studies of rhIL-7 in HIV-infected or refractory cancer patients revealed that the relative frequency of regulatory T cells (T_reg_) expressing low-level CD127 was reduced due to IL-7-induced expansion of conventional T-cells [24, 25]. Consistent with previous studies, the ratio of CD4^+^ and CD8^+^ T cells to regulatory T cells on day 21 after GX-I7 administration compared to baseline showed an increasing trend, and the increase was statistically significant in the 240–480 and 1200–1700 ug/kg groups (Fig. 3f). Figure 3g shows the average fold-change of CD4^+^ and CD8^+^ T cell subtypes at day 21 after GX-I7 administration compared to the baseline. While all CD4^+^ and CD8^+^ T cell subsets, including naïve, central memory (CM), and effector memory (EM) T cells, showed significantly increased numbers after GX-I7 administration, notable increases in the number were observed for naïve CD8^+^ and CD4^+^ T cells.

**Fig. 4:**
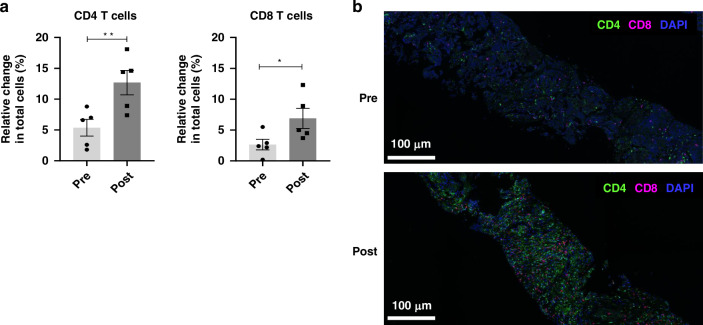
T cell subsets evaluation by Multiplex-IHC for tumour microenvironments at baseline (pre) and GX-I7 treatment (post) from solid tumour patients. All tissue samples were biopsied at the metastasized site, except for one pre-dose sample from one subject. **a** The percentages of infiltrating CD4^+^ and CD8^+^ T cells in the whole tumour samples were higher in post specimens. **b** Representative Multiplex-IHC image of pre-treatment (upper) and post-treatment (lower) in biopsied specimens. Scale bars: 100 μm for IHC and DAPI (blue) were used for nuclear staining. *p < 0.05, **p < 0.01 versus baseline (Day 0) group by Paired t-test.

Pre and post-treatment tumour biopsies were collected from five patients who consented to the intervention to evaluate changes in the number of tumour-infiltrating lymphocytes (TILs). Multiplex IHC was used in this study, and all biopsies were collected in the third week after the first injection or the second week after the second injection of GX-I7. A statistically significant increase in CD8^+^/CD4^+^ TILs was observed after GX-I7 treatment (720 µg/kg group, 1 patient; 1200 µg/kg group; four patients) (Fig. 4a). Figure 4b shows a representative image of tumour tissue from a patient treated at 1200 µg/kg, demonstrating increased CD4^+^ and CD8^+^ T cell infiltration at week 3 compared to baseline.

## Discussion

GX-I7 was shown to be safe and well tolerated at dosed up to 1200 µg/kg, given at intervals of 3- or 6-weeks. Tolerability and safety were confirmed in GX-I7 single subcutaneous and intramuscular dose-escalation tests in healthy subjects [23]. This clinical trial was conducted to confirm the safety and tolerability of GX-I7 in patients with locally advanced, recurrent, or metastatic incurable solid tumours. The most common adverse event and adverse drug reactions in all dose groups were injection site reactions, which were not severe and were also observed following the administration of other cytokine products [26]. MTD and RP2D were confirmed to be 1200 µg/kg based on the dose-limiting toxicity in each GX-I7 dose group. The DLT that occurred was one subject with grade 3 or higher hypersensitivity in the 1700 µg/kg group. Since the safety and tolerability of GX-I7 up to 1200 µg/kg have been established in patients with chemorefractory solid tumours, it is expected that it can be safely administered in combination with other anticancer drugs. For efficacy analysis, anti-tumour activity was assessed in 32 patients who received at least one dose of GX-I7 and completed one cycle of treatment with measurable disease. Six patients (18.75%) showed stable disease (SD), and the median progression-free survival (mPFS) was 5.69 weeks (95% CI:5.13–5.98 weeks), with similar mPFS between groups. GX-I7 is a safe and effective T-cell amplifier. Although the primary objective of the study was safety and the number of patients enroled was low, GX-I7 monotherapy showed a minimal clinical benefit, with six patients having stable disease, indicating the need for further studies, in monotherapy or in combination with other immunotherapies, to establish its clinical efficacy in larger trials.

The PK profile provided evidence that GX-I7 is a long-acting IL-7 with a dose-dependent increase in systemic exposure, as indicated by C_max_ and AUC_last_. PD analyses demonstrated that GX-I7 is capable of increasing the absolute lymphocyte counts (ALC), CD4^+^ and CD8^+^ T cells in a dose-dependent manner, but does not affect B cell counts. The increase in ALC, as assessed by ΔE_max_ and ΔAUEC, was positively correlated with systemic exposure to GX-I7, as indicated by C_max_ and AUC_last_ (Supplementary Fig. S1). In five available paired biopsies, GX-I7 also showed the ability to increase the number of tumour-infiltrating lymphocytes (TILs) of the CD4 and CD8 subtypes. ADA emergence was observed regardless of the dose and dosing interval, but it did not have an impact on the safety of GX-I7. The final RP2D was determined to be 1200 µg/kg based on the maximum observed change in ALC, used as a pharmacodynamic marker, within a dose range that demonstrated safety and tolerability. The increase in Ki67 expression and downregulation of CD127 provided evidence that the increase in ALC and T cells is due to the direct mechanism of action of GX-I7. Administration of GX-I7 resulted in an improvement in the CD4^+^ T cell/T_reg_ and CD8^+^ T cell/T_reg_ ratios, generating a favourable environment for enhanced immune activity.

In this study, most patients received at least two or more doses of GX-I7 at 3-week intervals. However, starting from the second repeated dosing, there was no observed increase in proliferation markers such as Ki67 in T cells and ALC (Supplementary Fig. S2a). Based on these results, GX-I7 was administered at 6-week intervals at a dose of 1200 µg/kg in the expansion phase. As shown in Supplementary Fig. S2b, the levels of Ki67^+^CD4^+^ and Ki67^+^CD8^+^ T cells, as well as the ALC level (although data from only one patient was available), increased in the blood following repeated dosing of GX-I7 at 6-week intervals. It was not possible to obtain long-term follow-up results of repeated dosing, as most of the patients participating in this clinical trial had late-stage cancer with very short life expectancies. For this reason, an animal study was conducted as a supportive measure in normal cynomolgus monkeys, and it showed that when the monkeys were repeatedly administered GX-I7 at a dose of 3 mg/kg with intervals of 6, 9, and 12-weeks, the proliferation markers and ALC showed a tendency to increase when administered at intervals of 6-week or longer (Supplementary Fig. S3). Therefore, the dosing interval can be considered an important factor that may affect the PD profile of GX-I7. Subsequent studies with GX-I7, administered with dosing intervals of 4, 6 weeks or longer, will be performed. ADA occurs in more than 90% of patients after receiving a dose of GX-I7, which is consistent with previous findings in healthy adults. In Phase 1 clinical trials in healthy volunteers, ADA was observed in most patients (75–100%) approximately 10 days after GX-I7 administration, but most of these antibodies disappeared within 330 days of follow-up.

In the present study, repeated administration of GX-I7 at 6-week intervals demonstrated an increase in proliferation markers, despite the presence of ADA. Similarly, in the monkey study, repeated administration of GX-I7 for 6 weeks or longer resulted in increased expression of proliferation markers, even in the presence of ADA (Supplementary Fig. S3, Supplementary Table S3). These results indicate that ADA production does not affect the ability of GX-I7 to increase ALC; however, further studies involving a larger number of patients with longer treatment durations are warranted to confirm these findings. The main pharmacodynamic response of GX-I7 in biological systems is an increase in ALC. However, it is still under investigation whether this GX-I7-related boost in ALC directly leads to increased TIL and mediates clinical efficacy. In the present study, as part of this investigation, multiplex IHC analysis was performed on a small number of patients whose tumours were biopsied, and an increase in CD4^+^ and CD8^+^ T cells was observed within the tumour tissues at 3 or 5 weeks after GX-I7 administration. Thus, GX-I7 as a T-cell amplifier provides a unique opportunity for immuno-oncology combination strategies by reconstituting persistent T-cells.

This study is limited by its small sample size and the inclusion of multiple cancer types. Further evaluation of GX-I7 in tumour environments should be conducted through larger-scale clinical trials.

In conclusion, these findings support the use of GX-I7 as a safe and effective T cell-amplifying agent for correcting T cell deficiency. Studies are ongoing to evaluate the potential clinical efficacy of GX-I7 in combination with other anti-cancer agents. While novel combinations with GX-I7 may have the potential to improve the tumour microenvironment and enhance the efficacy of ICI and other anti-cancer treatments, further data is needed to fully support these outcomes.

## Supplementary information

Supplemental material

## Data Availability

The data generated or analysed during this study contain individual-level clinical information and are not publicly available due to patient privacy and ethical restrictions. However, de-identified data may be made available from the corresponding author upon reasonable request and with appropriate institutional approvals.
